# A Novel Mobile Health Tool for Home-Based Identification of Neonatal Illness in Uganda: Formative Usability Study

**DOI:** 10.2196/14540

**Published:** 2019-08-15

**Authors:** Madison Vanosdoll, Natalie Ng, Anthony Ho, Allison Wallingford, Shicheng Xu, Shababa Binte Matin, Neha Verma, Azadeh Farzin, W Christopher Golden, Youseph Yazdi, Peter Waiswa, Alain Labrique, Soumyadipta Acharya

**Affiliations:** 1 Center for Bioengineering Innovation and Design Department of Biomedical Engineering Johns Hopkins University Baltimore, MD United States; 2 School of Medicine Johns Hopkins University Baltimore, MD United States; 3 Pediatrix Medical Group Rockville, MD United States; 4 Global mHealth Initiative Johns Hopkins University Baltimore, MD United States; 5 School of Public Health Makerere University Kampala Uganda; 6 Bloomberg School of Public Health Johns Hopkins University Baltimore, MD United States

**Keywords:** neonatal, community health workers, maternal, Uganda, World Health Organization, smartphone app, digital health, mobile health, telemedicine

## Abstract

**Background:**

While early identification of neonatal illness can impact neonatal mortality rates and reduce the burden of treatment, identifying subtle clinical signs and symptoms of possible severe illness is especially challenging in neonates. The World Health Organization and the United Nations Children’s Fund developed the Integrated Management of Neonatal Childhood Illness guidelines, an evidence-based tool highlighting seven danger signs to assess neonatal health. Currently, many mothers in low-resource settings rely on home visits from community health workers (CHWs) to determine if their baby is sick. However, CHWs visit infrequently, and illness is often detected too late to impact survival. Thus, delays in illness identification pose a significant barrier to providing expedient and effective care. Neonatal Monitoring (NeMo), a novel neonatal assessment tool, seeks to increase the frequency of neonatal screening by task-shifting identification of neonatal danger signs from CHWs to mothers.

**Objective:**

This study aimed to explore the usability and acceptability of the NeMo system among target users and volunteer CHWs by assessing ease of use and learnability.

**Methods:**

Simulated device use and semistructured interviews were conducted with 32 women in the Iganga-Mayuge districts in eastern Uganda to evaluate the usability of the NeMo system, which involves a smartphone app paired with a low cost, wearable band to aid in identification of neonatal illness. Two versions of the app were evaluated using a mixed methods approach, and version II of the app contained modifications based on observations of the first cohort’s use of the system. During the posed scenario simulations, participants were offered limited guidance from the study team in order to probe the intuitiveness of the NeMo system. The ability to complete a set of tasks with the system was tested and recorded for each participant and closed- and open-ended questions were used to elicit user feedback. Additionally, focus groups with 12 CHWs were conducted to lend additional context and insight to the usability and feasibility assessment.

**Results:**

A total of 13/22 subjects (59%) using app version I and 9/10 subjects (90%) using app version II were able to use the phone and app with no difficulty, despite varying levels of smartphone experience. Following modifications to the app’s audio instructions in version II, participants’ ability to accurately answer qualitative questions concerning neonatal danger signs improved by at least 200% for each qualitative danger sign. All participants agreed they would trust and use the NeMo system to assess the health of their babies. Furthermore, CHWs emphasized the importance of community sensitization towards the system to encourage its adoption and regular use, as well as the decision to seek care based on its recommendations.

**Conclusions:**

The NeMo system is an intuitive platform for neonatal assessment in a home setting and was found to be acceptable to women in rural Uganda.

## Introduction

### Background

Each year, 2.5 million newborns die in the first 28 days of life, with 80% of these deaths occurring within the first seven days of age [1]. In resource-limited settings in low- and middle- income countries (LMICs), a majority of these deaths occur within homes largely due to preventable causes such as pneumonia, sepsis, and other illnesses [2,3]. The neonatal mortality rate in Uganda is 26 per 1000 live births [4]. In rural districts in eastern Uganda where 34 in every 1000 newborns die in their first month of life, neonates are even more vulnerable [3]. Many of these deaths could be averted by timely identification and referral to treatment [5,6].

While as many as 84% of neonatal infection-related deaths could be prevented with available interventions, identifying subtle clinical signs and symptoms of severe illness is especially challenging in neonates [5]. Thus, delays in illness identification pose a significant barrier to providing expedient care [6-8]. An analysis of 64 neonatal deaths in eastern Uganda showed that the highest contributing delays to newborn intervention were challenges in problem recognition and in the decision to seek care (50%), with a median time of 3 days from onset of illness to seeking care outside the home [3].

In order to improve recognition of neonatal illness, the World Health Organization (WHO) has developed the Integrated Management of Neonatal and Childhood Illness (IMNCI) guidelines [9-11]. Studies have shown the following seven danger signs predict severe illness in neonates: (1) difficulty feeding; (2) convulsions; (3) lethargy; (4) chest indrawing; (5) respiratory rate of 60 breaths per minute or more; (6) temperature above 37.5°C; or (7) temperature below 35.5°C [9]. Presentation of a danger sign indicates the need for immediate medical attention [9,12]. Although effective identification of these signs at the community level can intercept illness and incite care-seeking behavior capable of impacting child mortality, the tools and training needed to assess indicators of illness are lacking in low-income settings [6,13-17].

Currently, rural health care systems in Uganda and other LMICs rely on village health teams (VHTs) of volunteer community health workers (CHWs) to visit mothers at home to provide postnatal care. While women visited by CHWs are 87% less likely to lose their newborns, these volunteers are often overburdened, and limited by both availability and bandwidth [18]. Thus, CHWs are often unable to complete the three postnatal home visits recommended by the WHO during the critical first week of life. As few as 5% of Ugandan newborns receive a CHW visit in the first 48 hours of life and sick infants are often identified too late to impact survival [19]. Task-shifting neonatal assessment from CHWs to mothers is a promising strategy to reduce neonatal mortality [6,7,20]. While the aforementioned danger signs can have a sensitivity of 85% and specificity of 75% when used by a trained primary care worker to identify neonatal illness, unassisted maternal recognition of these symptoms in community settings has failed to exceed a sensitivity of 20-24% [7,9,21,22].

### The NeMo System: A Low-Cost Technology Platform for Assessment of Neonatal Danger Signs

The opportunity to improve identification of neonatal illness in home settings was identified by a team of faculty and biomedical engineering graduate students at the Johns Hopkins University Center for Bioengineering Innovation and Design (CBID) and School of Public Health, based on over 18 years of community research in rural Bangladesh [23-25]. Engineering design teams had the opportunity to confirm these population research findings through community and rural health system immersion in Iganga, Uganda. Primary observations from ethnographic research conducted in Iganga highlighted delays in identification of neonatal illness due to a systemic reliance on overburdened CHWs. Teams also visited rural communities in Kenya and Bangladesh and observed similar patterns. The design teams identified the need for a low-cost technology to regularly, objectively, and accurately assess the seven previously mentioned IMNCI danger signs during the first week of life to expedite detection of neonatal illness in LMICs. By empowering care providers within the family unit (ie, mothers) to detect signs of illness, the team hypothesized that timely care-seeking behavior could be triggered when it otherwise might not be. Neonatal Monitoring (NeMo), a proposed solution to the need for earlier identification of neonatal illness, is a two-part system designed to empower mothers to effectively assess the seven validated IMNCI danger signs.

**Figure 1:**
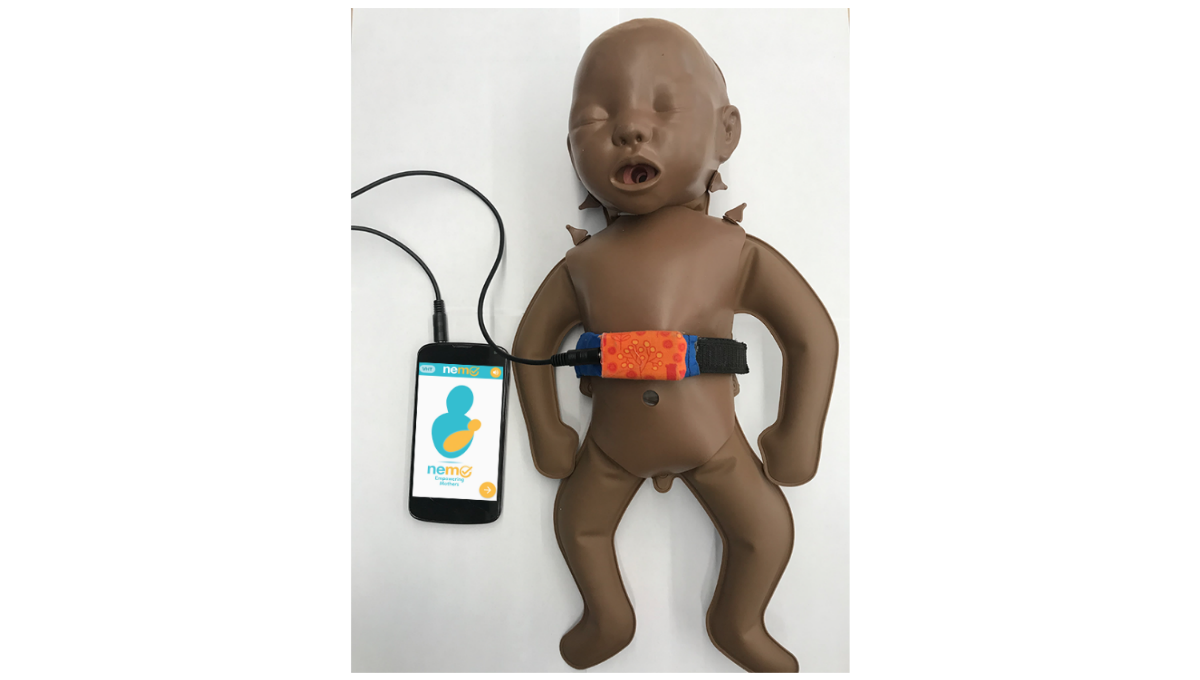
The NeMo (Neonatal Monitoring) band goes around the neonate’s abdomen to acquire respiratory rate and temperature data. The data is then transmitted via an audio cable and processed in the NeMo app, where it is then integrated with mothers' responses regarding qualitative danger signs to provide a recommendation on whether a mother should seek care for her neonate.

**Figure 2:**
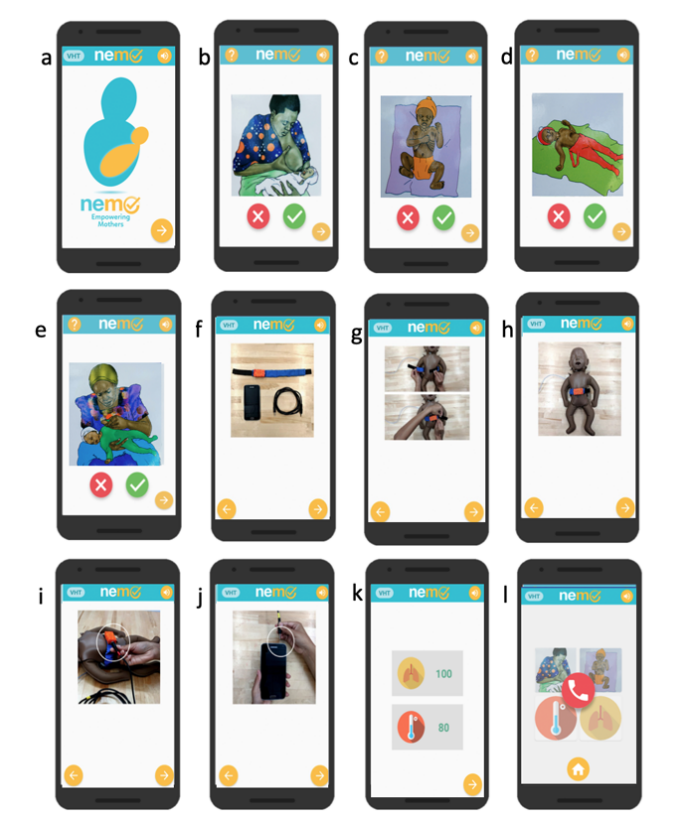
Illustration of the NeMo app, with qualitative danger sign assessment screens: a) home screen; b) difficulty breastfeeding; c) chest indrawing; d) convulsions; and e) lethargy. The mother can select the X or check symbols to respond as to whether her child is sick or healthy. Instructions on how to place the band are shown in f-j. Respiratory rate and temperature are displayed in k). Danger signs detected, and need to call a CHW are shown in l. NeMo: Neonatal Monitoring; CHW: community health worker.

The NeMo system consists of a novel, wearable sensing band (the NeMo band) and a low-cost smartphone preloaded with the custom NeMo app (Figure 1). The NeMo band fastens around the neonate’s abdomen using a generic hook-and-loop fastener like Velcro (herein after referred to as velcro). It is equipped with sensors to measure temperature and respiratory rate, which are difficult to assess without appropriate training or tools. These sensors are housed in a small plastic enclosure which must be placed centrally on the neonate’s abdomen (in between the nipples and midway between the umbilical cord and the nipples) (Figure 1). Information from the respiratory and temperature sensors is transmitted to the phone for processing via a standard audio cable which plugs into the audio jack on the phone and via an identical jack embedded into the enclosure on the NeMo band. The app uses audio and visual cues in the local language to enable any mother, regardless of literacy, to assess the qualitative danger signs and place the NeMo band (Figure 2). By responding to a series of interactive audio prompts concerning her newborn’s health, the mother is asked to indicate whether or not her baby has any danger signs by selecting either a check or X on the app interface (ie, “Has your baby been refusing to breastfeed? Press check if yes, press X if no.”). The app does not require internet connectivity. By synthesizing the results from the quantitative and qualitative assessments, NeMo alerts mothers of any danger signs detected and advises users if the newborn requires medical attention.

When using the system, the mother is first taken to a home screen (Figure 2a), which introduces the navigational features of the app. The mother is then guided through individual screens which ask her to assess the presence of the four qualitative danger signs: difficulty breastfeeding (Figure 2b), chest indrawing (Figure 2c), convulsions (Figure 2d), and lethargy (Figure 2e). The app then uses audio instructions and images to walk the mother through each step of properly placing and securing the band around her baby and connecting the band to the phone with the audio cord (Figure 2f-j). Once positioned around the neonate’s abdomen, the NeMo band measures temperature and respiratory rate (Figure 2k). Following the quantitative assessment, the app displays representative images of any danger signs detected and, if indicated, produces a red phone icon to prompt the mother to call a CHW for further assessment and help (Figure 2l). The mother only needs to press the icon to initiate the phone call.

NeMo is based on a CHW-coordinated, phone-sharing framework. In this model, mothers are provided a smartphone by a CHW for the week following birth and purchase their own low cost, wearable band. After the first seven days of the newborn’s life, the phone will be returned to the CHW and the band discarded to prevent the spread of infection between newborns. By making both this technology and education to support clinical evaluation accessible in community settings, NeMo could empower mothers to assess neonatal illness at home and without support. While evidence supporting the acceptability of phone use among CHWs is abundant, evaluation of a community phone-sharing model and maternal smartphone use to enable neonatal mobile health (mHealth) interventions remains lacking [26-28]. Therefore, for any digital health intervention targeting mothers in LMICs directly, there is a need to validate acceptability and usability among target users.

### Objectives

While several studies have established the utility of the seven danger signs in identifying neonates in need of care, the ability of mothers to utilize a simple, digital health strategy to guide effective assessment of these signs has not been established [9,12]. Thus, this study was designed to evaluate the usability and acceptability of the NeMo smartphone app and wearable sensor among both women of child-bearing age and CHWs in the Iganga-Mayuge districts of Uganda. Further, this study seeks to evaluate women’s and CHWs’ perceptions of the phone-sharing model and their willingness to use the NeMo device to evaluate the health of their neonates.

## Methods

### Overview

This study was undertaken in eastern Uganda in the Iganga-Mayuge districts, over the course of two weeks, in five villages. These districts were selected for their representation of rural Uganda, offering socioeconomic diversity and corresponding literacy rates [3,29,30]. Fieldwork was supported by Uganda Development and Health Associates (UDHA), which has a regional headquarters based in Iganga. Villages in these districts were visited to capture perceptions of the usability and acceptability of the NeMo system within populations at high risk for vulnerability [29,31]. For both study participants and CHWs, a convenience sampling strategy was utilized. Village leaders randomly selected female citizens from their village within reproductive age (18-49 years old) to be interviewed. CHWs were randomly selected via their connection with UDHA.

This study was conducted following protocols approved by the Johns Hopkins Homewood Institutional Review Board (IRB00005144) and by the Institutional Review Board of Makerere University School of Public Health (MakSPH IRB) in Uganda. Written consent was obtained from all parties including study subjects and CHWs before each interview and was available in Lusoga and English.

### Simulated Use of the NeMo System

The qualitative study consisted of semistructured interviews with all women (n=32) and volunteer CHWs (n=12). With each subject, a preliminary structured interview was conducted to collect demographic information. Subsequently, each subject participated in a verbally posed simulated scenario to assess the usability of the NeMo system. Subjects utilized the NeMo app preloaded onto an Android smartphone, along with a lookalike prototype of the NeMo device. The steps of the simulation were carried out on a NeoNatalie inflatable simulator (Laerdal Medical, Norway)**.** A comprehensive list of the simulated tasks can be found in Table 1.

The usability of the app interface was measured based on the subjects’ ability to navigate through the app and accurately answer the qualitative questions based on the posed scenarios. Additionally, the ability of each participant to position the device on the NeoNatalie’s chest and correctly insert the audio cord into both audio jacks was used as a measure of usability. The usability assessment was further rounded out by a series of closed- and open-ended questions, followed by a series of questions answered using a Likert scale.

Subjects were shown two alternative versions of the band and two alternative versions of the app. They were then asked to indicate their preferences based on their perceptions of ease of use, helpfulness, and safety.

This study was conducted in two phases, reflecting version I and version II of the app interface. The first phase (n=22) followed the methods detailed above and used app version I. The second phase (n=10) utilized version II of the app, which was updated based on qualitative and quantitative results from phase one. All study methods remained consistent between the two phases of the study. Women enrolled in the study were only given either version I or version II of the app.

Statistical significance was determined using a Fisher’s Exact Test to determine subjects’ association with a green check and red X and between mothers utilizing each version of the app. A Likert scale was used to gauge subjects’ opinions on the NeMo system and converted to a composite score (1=Strongly Disagree, 5=Strongly Agree). All other responses were analyzed using descriptive statistics.

**Table 1:** Outline of study tasks and questions asked regarding the subjects' experience using the NeMo system.

Task #	Type	Description
1	Consent	Study team members obtained written consent from subject.
2	Assessment of symbol comprehension	Before the subject was introduced to the phone app, she was shown printed images of a green check and a red X and asked to point to the image that meant “yes” or “no,” respectively.
3	Ability to use smartphone	The subject was shown how to unlock the phone and open the app, but not how to navigate through the NeMo^a^ app. She was then asked to unlock the smartphone device, open the app, and begin clicking through it, following the audio prompts. If the subject appeared to be stuck on a screen, she was prompted to replay the audio cue rather than guided on how to proceed, and her hesitation was noted by the study team. Specific tasks assessed included: unlocking the phone opening the app navigating through the app from homescreen
4	Answering questions regarding qualitative danger signs	Before the subject answered each of the app’s questions regarding qualitative danger signs, the interviewer stated whether the NeoNatalie was hypothetically afflicted with the relevant condition. For example, the interviewer would state, “The baby is convulsing,” or, “The baby is healthy; she is not convulsing,” in reference to the NeoNatalie. The subject was then asked to respond to the app’s questions based on the posed scenario given by the interviewer.
5	Device placement & audio cord insertion	The subject’s ability to correctly insert the audio cord into the device and the smartphone audio jack, as well as her ability to place the band in the center of the simulation mannequin’s chest when guided only by the app’s audio and visual cues was evaluated. Specific tasks assessed included: device placement on the simulation mannequin successful connection of audio cord into the device successful connection of audio cord into the phone
6	User feedback	The subject was asked a series of closed- and open-ended questions, followed by a series of questions answered using a Likert scale to probe her perceptions of the NeMo system. Questions covered the following themes: Would you use this device on your baby? Were there any danger signs mentioned on the phone that you did not understand? How much, if anything, would you be willing to pay for the device?
7	Intent to act	To qualitatively assess subjects’ intent to act on NeMo’s recommendation on whether or not to seek care, the subject was asked to respond to two hypothetical questions: If the device says your baby is sick, but you think your baby is healthy, what would you do? If the device says that your baby is healthy, but you believe your baby is sick, what would you do?
8	Alternative band embodiments	The subject was shown two additional versions of the NeMo band with different fastening mechanisms and asked to practice placing them on the NeoNatalie. She was then asked to answer open-ended questions concerning which embodiment she felt was safest, easiest to use, and which one she would be most likely to use on her own baby.
9	Alternative app embodiments	The subject was shown two additional versions of the NeMo app and asked to answer similar questions pertaining to ease of use and helpfulness for each version.

^a^NeMo: Neonatal Monitoring

### Community Health Worker Focus Groups

Three focus groups, facilitated by 2-3 study team members and a translator, were conducted with groups of 3-5 CHWs. A total of 12 CHWs participated in focus group discussions. Each focus group lasted approximately 60 minutes, with question themes including mothers’ ability to use the system, the feasibility of a CHW-lead training and sensitization initiative, and the acceptability of a community phone-sharing model. Table 2 details key topics discussed with CHWs.

Five English-speaking study team members were trained to conduct the interviews and were provided interview guides. The majority of the interviews were conducted in Lusoga through the aid of two translators, however, some study participants and CHWs were able to understand and respond to the interview questions in English. All interviews were video recorded with participant consent.

**Table 2:** Outline of themes explored in focus group discussions with community health workers (CHWs).

Theme		Examples
1	CHW background	Personal experience, training, responsibilities Current practices for neonatal assessment
2	Training mothers	Current practices providing antenatal care Danger sign recognition training for mothers
3	Acceptability of technology	Smartphone experience Comfort level training mothers on smartphone
4	Business model validation	Perception of phone sharing model Willingness to coordinate band sales and phone sharing

## Results

### Study Population Demographics

The study cohort consisted of 32 women from 5 different villages across the Iganga-Mayuge districts. Women were screened to be of child-bearing age or older, so they ranged from 19 to 44 years of age, with an average age of 27. They had each given birth to a range of 0-6 children, averaging 2.6 children.

Of all the subjects, 30/32 (94%) either owned or had access to a cellphone. However, the majority of these were not smartphones, with only 10/32 subjects (32%) having previously used a smartphone. In acknowledgement of the importance of neonatal danger sign recognition, the Ugandan government has mandated that danger signs be taught in antenatal care education. All subjects enrolled in phase two of the study received antenatal and postnatal education. Two of the women interviewed in phase two of the study did not have children and were not included in this survey analysis. Despite the expectation of proficiency in identifying signs of illness following this training, subjects' retention was low: only 3 of the 8 mothers (37.5%) recognized fever as a danger sign. Furthermore, only 3 mothers were able to correctly identify one additional danger sign (Table 3). These results emphasize the gap in mothers’ education and knowledge surrounding the assessment of neonatal illness and highlight the potential value of an effective tool to guide identification of danger signs in the home setting.

**Table 3:** Mothers’ recall of neonatal danger signs in study phase two (n=8).

Danger signs	Mothers, %
Fever	37.5
Chest indrawing	12.5
Increased respiratory rate	12.5
Lethargy	12.5
Hypothermia	0
Refusing to breastfeed	0
Convulsions	0

### Assessment of Symbol Comprehension

Of all the subjects, 30/32 (94%; *P*<.001) correctly identified the green check to mean yes and the red X to mean no, while two women were unfamiliar with this symbology. These two women thus had the meaning of each symbol explained to them.

### Ability to Use Smartphone

Of the 22 subjects that had never used a smartphone before, 18 (82%) could unlock the phone and open the app without additional guidance and 20 (91%) could do at least one or the other without additional guidance.

### App Version I

#### App Navigation

**Figure 3:**
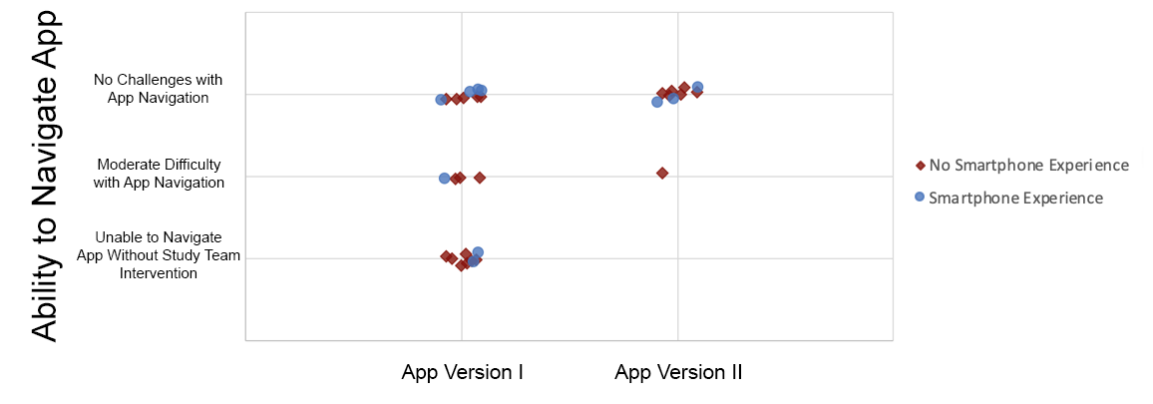
Comparison of mothers' ability to navigate version I and version II of the app based on previous smartphone experience. Mothers using version II of the app showed a significant improvement in app navigation (*P*=.02).

In total, 13/22 subjects (59%) enrolled in phase one required no external prompting or direction from the interviewer or translator to navigate from the homescreen to begin the assessment (Figure 3). A lack of specificity in the verbal cues indicating what button should be clicked to proceed often prevented subjects from navigating through the app.

#### Answering Questions Regarding Qualitative Danger Signs

Across both versions of the app, all subjects responded that there were no danger signs introduced by the app they had not understood. Although this was the first time two subjects had heard about chest indrawing, 28/32 women (88%) responded that they were confident or very confident in their ability to identify those danger signs in their own infants. However, this study only assessed a subject’s ability to utilize the NeMo system to input information regarding their newborn’s health. Determining their accuracy in identifying qualitative danger signs is beyond the scope of this study. All 32 subjects responded that the voice directions in the app were intuitive and easy to understand.

**Figure 4:**
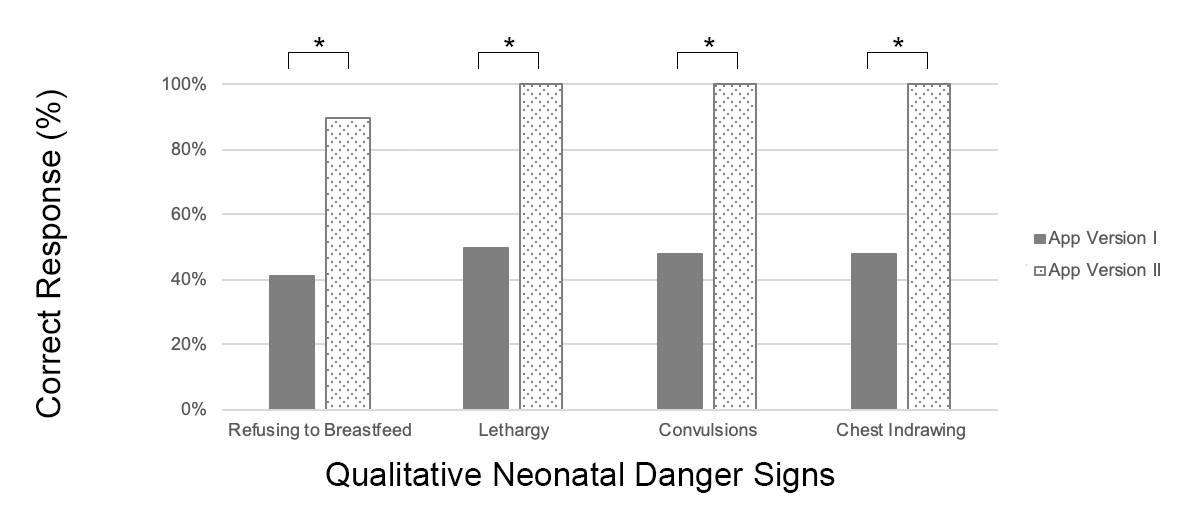
Comparison of mothers' ability to correctly respond to verbal scenarios using app versions I and II. Their ability to answer qualitative questions significantly improved across all four danger signs. * indicates *P*<.05.

In version I of the app, only one subject was able to correctly answer all 4 of the qualitative questions based on the posed scenarios and only 6/22 subjects (27%) were able to correctly answer 3 of the 4 questions (Figure 4).

#### Device Placement & Audio Cord Insertion

As correct placement of the NeMo device on the abdomen of the neonate is critical to obtaining accurate measurements for both respiratory rate and temperature, a subject’s ability to properly position the device around the NeoNatalie simulator using only the verbal instructions from the app was used as a measure of ease of use of the device. Using version I of the app, 18/22 subjects (82%) were able to properly position the device with only the verbal instructions and no prior instruction or training. Additionally, 20/22 subjects (91%) were able to successfully connect the audio cable to both the device and smartphone.

### Changes made Between App Version I and Version II

Subjects stuck on the homescreen in version I often attempted to press different areas on the screen (including the check mark in the NeMo logo) in an attempt to advance to the next screen. To avoid this challenge, in version II the team added more detailed audio instructions explaining the location of the button to continue to the next screen.

As a result of the poor ability of subjects to answer qualitative questions based on the simulated scenarios using version I of the app, the team evaluated what factors might be at fault. One translator suggested that subjects might be responding in reference to their own infants rather than to the posed NeoNatalie scenarios. The team also hypothesized that questions were being posed in a misleading way based on the positive and negative connotations of the check and X symbols. Originally, the app instructed subjects to press the green check if the baby had the danger sign (“Yes, my baby has this danger sign”) and the red X if the baby did not have the danger sign (“No, my baby does not have this danger sign”). However, it was suggested that subjects might not intuitively associate a green check with illness and a red X with a healthy baby. Thus, in app version II, the team rephrased the app audio, instructing subjects to press the green check if their baby was healthy and did not have the sign and the red X if their baby was sick and did have the sign.

Finally, while most women using version I of the app were able to correctly place the device, the NeMo app’s audio cues were updated to further assist subjects with device placement. Greater detail was given about anatomical structures on the newborn, specifying the position of the device relative to the nipples and umbilical cord in version II.

### App Version II

#### App Navigation

All subjects utilizing version II of the app, regardless of previous smartphone exposure, were able to turn on the smartphone and successfully navigate through the app without intervention by the study team. Figure 3 illustrates a comparison between the different app versions and subjects’ exposure to smartphones.

For both versions of the app, subjects were assessed on ease of app navigation based on observed hesitation and need for further explanation and prompting by the study team. The introduction of version II resulted in significant improvement in subjects’ ability to navigate the app interface, as assessed by comparing the number of women that were able to navigate the app without any challenges to those who could not (*P*=.02).

#### Answering Questions Regarding Qualitative Danger Signs

Using version II of the app, 9/10 (90%) of the remaining subjects correctly answered all four of the qualitative questions based on the posed scenarios. The remaining subject answered three of the four questions correctly. This modification resulted in significant improvement in subjects’ ability to respond to each of the qualitative danger sign questions using the NeMo app interface: breastfeeding (*P*=.02), lethargy (*P*=.006), convulsions (*P*=.005), and chest indrawing (*P*=.005). A comparison between subjects’ performance using version I and version II of the app is shown in Figure 4.

#### Placement and Audio Cord Insertion Comparison

Using improved audio instruction in app version II, all subjects were able to correctly position the band on the NeoNatalie. While these results showed improvement from the 18/22 subjects (82%; *P*=.28) that were able to place the band correctly and the 20/22 subjects (91%; *P*>.99) that were able to connect the audio cable properly, these changes were not found to be statistically significant.

### Perception of Usability and Acceptability

#### Ease of Use and Learnability

**Figure 5:**
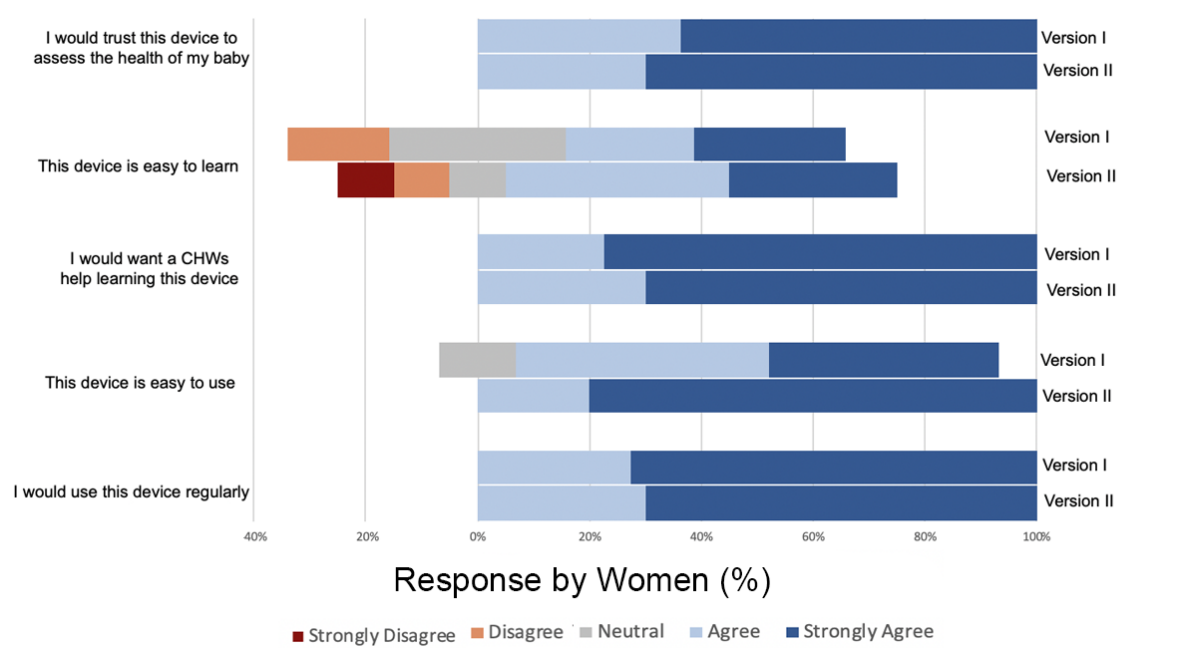
Mothers' responses to statements regarding perception of the NeMo device scored on a Likert scale. CHW: community health worker.

Each subject was asked about her perception of the system’s ease of use, and they responded using a Likert scale. A comparison between responses from women enrolled in phase one and phase two was carried out, but their responses were not found to be significantly different. Responses provided by subjects in both phases of the study are displayed in Figure 5. Using a composite scoring system (Strongly Agree=5, Strongly Disagree=1), the device had an ease of use score of 4.34 (median 5, interquartile range [IQR] 1) and a learnability score of 3.56 (median 4, IQR 2). In addition, all subjects surveyed agreed or strongly agreed they would like a CHW’s help learning how to use the device, giving a composite score of 4.75 (median 5, IQR 1).

#### Trust in Technology and Intent to Use

All subjects surveyed indicated they agree or strongly agree they would trust NeMo to assess the health of their baby, giving a composite score of 4.66 (median 5, IQR 1). Additionally, all subjects agree or strongly agree that they would use NeMo regularly, giving a composite score of 4.72 (median 5, IQR 1).

#### Intent to Act

A total of 29/32 subjects (91%) stated they would take their baby to a health care facility if the device told them their baby was sick, even if they believed the baby was healthy. Alternatively, 24/32 subjects (75%) responded that they would take their baby to a health care facility if they believed their baby was sick, even if the device told them their baby was healthy. These findings support subjects' trust in the device and the potential for NeMo to trigger care-seeking behavior, while also demonstrating women's agency in seeking medical attention even when NeMo does not signal illness.

#### Perception of Band Embodiments

**Figure 6:**
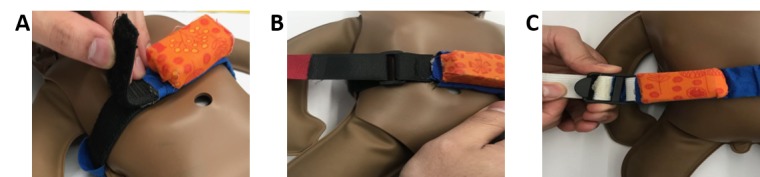
NeMo band embodiments: (A) velcro; (B) velcro-with-loop; (C) adjustable strap.

In addition to evaluating usability and acceptability of the current versions of the NeMo system and band embodiment, the team developed two additional bands to evaluate which method of fastening was most acceptable to study subjects. For each of the three band variations shown in Figure 6, subjects placed the band on the NeoNatalie simulator and then evaluated which band they liked best, found easiest to use, were most likely to use, and thought would be safest. The velcro band utilizes a press-seal–fixation mechanism, while the velcro-with-loop uses similar velcro fixation with the addition of a loop to aid adjustments of tightness. The adjustable strap is slid over the newborn’s lower half and utilizes a friction fastening mechanism that slides along the strap to tighten. Results reported by the cohort are displayed in Table 4. A majority of 22/32 subjects (69%) found the velcro-with-loop band to be their favorite. Similarly, 17/32 subjects (53%) considered the velcro-with-loop easiest to use, while 14/32 subjects (44%) preferred the velcro band.

**Table 4:** Subjects' perceptions of different band embodiments (n=32). All values are given as n (%).

Embodiment	Velcro, n (%)	Velcro-with-loop, n (%)	Adjustable strap, n (%)
Liked best	9 (28)	22 (69)	1 (3)
Easiest to use	14 (44)	17 (53)	1 (3)
Safest	11 (34)	13 (41)	8 (24)

#### Perception of App Embodiments

**Figure 7:**
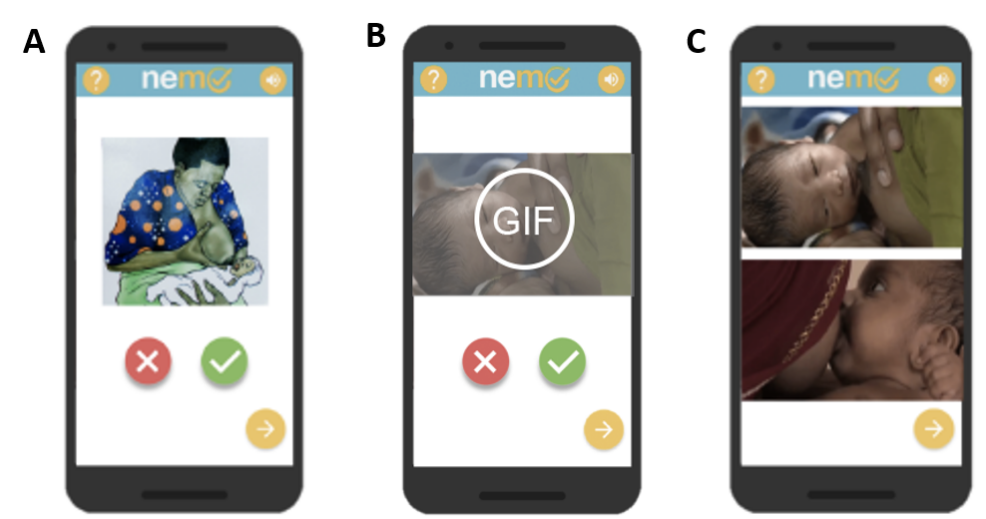
NeMo app embodiments: (A) illustrations; (B) animated GIF; (C) clickable pictures.

Subjects were also shown three different variations of the phone app and asked to rank which was the easiest to use, which was most helpful, and which one they liked the best (Figure 7). The distribution of their preferences among the app interfaces is displayed in Table 5. The new interfaces developed alongside the users were favored. Subjects indicated the animated graphic interchange format (GIF) app was the easiest, while the clickable pictures interface was the most liked and most helpful variation. 31 subjects responded to which app was easiest to use, and 30 subjects responded to which app was liked best and most helpful.

**Table 5:** Subjects' perception of different app embodiments. All values are given as n (%).

App interface	Illustrations, n (%)	Animated GIF^a^, n (%)	Clickable pictures, n (%)
Easiest	8 (26)	12 (39)	11 (35)
Liked best	8 (27)	10 (33)	12 (40)
Most helpful	4 (13)	15 (50)	11 (37)

^a^GIF: graphic interchange format.

### Amount Women are Willing to Pay for NeMo Device

**Figure 8:**
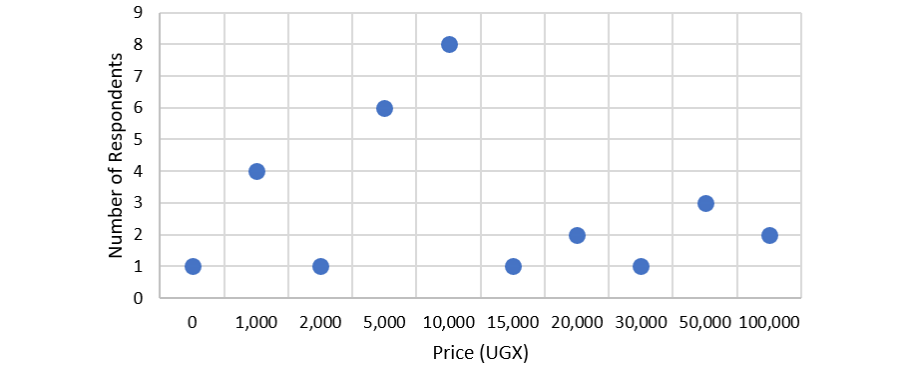
Distribution of mothers' willingness to pay for NeMo device.

In addition to assessing the usability of the band and the phone app, subjects were asked questions regarding the amount they would be willing to pay for the device. A total of 30/32 subjects (94%) were willing to make a one-time payment to purchase a NeMo band usable for the seven days following birth. The amount they were willing to pay varied between 1,000 to 100,000 Ugandan shillings (UGX), or approximately $0.27 to $27.00 United States dollars (USD). The median price was 10,000 UGX, or approximately $2.70, and the mean was 19,000 UGX, or $5.14. Figure 8 illustrates the distribution of prices subjects indicated they would pay for the NeMo band. While most subjects indicated they would be willing to pay for the device, participants were less receptive to the prospect of making a security deposit to borrow the smartphone. In total, 20/27 subjects (74%) indicated they would be comfortable making a security deposit they could collect after the device was returned.

### Insights from Community Health Worker Interviews

Three focus groups conducted with a total of twelve CHWs highlighted critical logistical considerations for the successful implementation of the NeMo system in Uganda. Through these conversations, it was confirmed that CHWs are expected to visit mothers three times during the first week of life. Additionally, CHW assessments of critical quantitative danger signs, including respiratory rate and temperature, are most often only qualitative in nature and are conducted by visually observing the baby’s breathing as well as touching the baby to determine temperature. Regarding environmental factors, CHWs were optimistic about mothers’ abilities to charge the phones, especially considering that most families own at least one phone and solar power is becoming increasingly more available in village homes.

#### Community Health Workers’ Perceptions of Mothers’ Ability to Use the NeMo System

CHWs were generally supportive of the NeMo system and believed that the device could help reduce neonatal mortality. However, a few participants expressed concern that some mothers might either lack the education to identify danger signs or opt to visit traditional birth assistants (TBAs) rather than a CHW or health facility. Despite these doubts, all twelve CHWs agreed they would be eager and willing to lead training programs in which mothers would be instructed in the use of the NeMo system.

#### Community Sensitization

The CHWs illustrated the importance of sensitizing the community to the utility and value of the device in order to ensure regular use and to encourage familial agreement to purchase the device. This community sensitization will also be paramount in determining whether or not mothers act on the suggestions of the NeMo system regarding their decision to seek care. Furthermore, it may dictate the safety of the phones in the community, reducing the likelihood of other community members attempting to steal them.

#### Payment for the System

After expressing concern about requiring mothers to pay for the sensing bands, numerous CHWs questioned whether the band could be used on multiple babies or for a duration longer than a week in order to give mothers greater incentive to purchase the device. A number of senior CHWs spoke of a poverty grading tool they utilized to determine the price point for a voucher that would cover the costs of pregnancy and postnatal care. A similar tool could be used to evaluate the cost of the NeMo system on a case-by-case basis.

#### Phone-Sharing Model

Focus group participants expressed hesitation over the phone-sharing model, claiming some mothers would lose the phone or that the phone was likely to be damaged. One CHW believed a phone-sharing model would not work because villagers would not understand who the phone belonged to and thus who was responsible for it. Again, emphasis was placed on the need to sensitize the community to the importance and utility of the device and phone-sharing model in order to gain acceptance. Because CHWs in Uganda are volunteer health care providers, it is frowned upon for them to accept money for their services. Thus, multiple CHWs rejected a proposed model in which CHWs would purchase the phones with their own money and sell the band above cost to recuperate the funds they spent on the phones. As reported by focus group participants, very few CHWs own smartphones. Thus, expecting these individuals to purchase one or more smartphones for their communities is likely unreasonable. For similar reasons, it was suggested that anything CHWs were responsible for selling to mothers (such as the NeMo sensing bands) needed to have an irremovable price tag so that there would be no concern that CHWs were potentially overcharging to make a profit.

## Discussion

### Principal Findings

While early identification of neonatal illness can impact neonatal mortality rates and reduce the burden of treatment, identifying subtle clinical signs and symptoms of possible severe illness is especially challenging in neonates. Thus, delays in illness identification pose a significant barrier to providing expedient care. Implementation of IMNCI and danger sign recognition have been shown to improve inequities in neonatal mortality and care-seeking behavior; however, the success of such interventions may be limited by the consistency of CHW follow-up and training [15,16,32]. Further, the current lack of sensitivity in maternal screening limits the feasibility of at-home danger sign screening [7]. The proposed NeMo system has the potential to empower mothers in community settings to assess their neonates’ health from home and without support. Further validation of mothers’ use during the newborn’s first week of life, behavior change catalyzed by triage information, and integration into the existing health care system must still be conducted.

When asked about their prior training in antenatal care, all subjects indicated they had received some antenatal education. However, the observed discrepancy between training and recollection of danger signs emphasizes the value the NeMo device might add as an educational tool and assessment aid.

Although many subjects had never used a smartphone before the study, the majority were able to unlock the phone and navigate through the NeMo phone app without external guidance. Improvements made in the app between version I and version II pointed to the importance of precise verbal instruction. Small changes to how questions were framed and how instructions were articulated, without any change to the visual appearance of the app, significantly increased subjects’ ability to correctly answer qualitative questions based on a posed scenario (Figure 4). In version II of the app, no subjects required prompting or further guidance from the study team to navigate the app, despite their unfamiliarity with smartphones prior to the study (Figure 3). This indicates sufficient verbal instruction and intuitive app design can overcome the barrier of introducing unfamiliar technology. Further, these results suggest lack of prior smartphone experience may not be a barrier to successful use of the NeMo system.

Many participants expressed their trust in the device’s evaluation, with agreement to the statement, “I would trust this device to assess the health of my baby,” receiving a score of 4.66 on the 5-point Likert scale. Women also indicated willingness to engage with the proposed system of phone-sharing. This acceptance suggests NeMo and other such frameworks may be acceptable in low-income, eastern Ugandan communities. While 29/32 subjects (91%) thought the device was easy to use, perceptions around learnability were not as conclusive. In addition to these results, unanimous desire for CHW support indicates the value of engaging CHWs in training programs and device management in order to successfully shift neonatal assessment to the home setting.

One challenge facing implementation of the NeMo system is creating trust in the device output sufficient enough to trigger behavior change and promote care-seeking. While care-seeking is a multifactorial and complex decision, preliminary questions were asked to gauge the subjects’ initial response to the NeMo system. In order to assess the ability to incite care-seeking, the team asked subjects how they would respond if the device said the baby was sick when they believed it was healthy. As 29/32 (91%) women indicated they would seek care from a CHW or hospital, this suggests that the NeMo device may be able to trigger a behavior change to reduce time to care for sick neonates. One potential risk posed by the device is mothers ignoring their own intuition and relying too heavily on NeMo. The results of this study indicated 24/32 subjects (75%) would take their baby to a facility if they thought it was sick, even if the device indicated it was healthy. This majority suggests most mothers retain autonomy in their decision making.

The ability of the NeMo device to improve care-seeking for neonates will rely heavily on the accessibility of the technology in the home setting for LMICs. In order to successfully transition assessment from CHWs to mothers, the smartphone and device must be available at a low enough cost to enable use in low-income households. In the eastern Ugandan districts where the study was conducted, subjects indicated a median price point of 10,000 UGX ($2.70) for the sensor. To improve accessibility of the smartphone, the study team developed a community phone-sharing framework in which CHWs would manage phones for all mothers in their village to borrow in the week following birth. Interviews with CHWs revealed concern about coordinating a phone-sharing network if the CHW is receiving payment directly, and some CHWs worried about the phones being lost or broken. However, focus groups also emphasized the ability of community sensitization to educate mothers on the value of the device so as to minimize these risks and increase likelihood of purchasing the device.

Despite the promising acceptability and usability results in this study and potential for improved maternal recognition to reduce neonatal mortality, the study revealed several barriers to implementing and scaling the NeMo system. One of these issues is the phone-sharing framework, which attempts to minimize the barriers to technology in rural, low-income regions by distributing phones on a community-by-community basis. However, in order to initiate the implementation of this phone-sharing system, government or private foundations will need to provide funding and support. Without their aid, the likelihood of the populations studied herein accessing this technology is low until smartphone penetration increases substantially. Although implementation of the NeMo system could significantly increase the frequency of neonatal screening, the device distribution system proposed herein would continue to require CHW interaction and support. Incorporation of distribution and training in existing antenatal care programs could enable this contact for many women. As access to antenatal care is limited in many areas, alternative intervention strategies centered around the opportunity for training and distribution around the time of birth may be employed to overcome barriers to CHW access. Further, while this study demonstrates promising results regarding perceived intent to act on a recommendation provided by the device, past researchers have found that barriers, including money and transportation, often bar mothers from following through with referrals to health facilities [3,33]. Even after a mother acts on NeMo’s recommendation to seek care, infrastructural or other barriers may prevent sick newborns from receiving the necessary treatments to improve neonatal mortality. This suggests initial intervention in a region with more robust infrastructure may foster a greater impact.

### Limitations

While the study was conducted in multiple subcounties and villages within the Iganga-Mayuge districts with a notably wide range of socioeconomic backgrounds, the limited sample size prevents greater generalizability of the study results. When posed hypothetical situations of neonatal illness during the simulated scenarios, a few subjects were noted to have responded about the condition of their own child instead of following the study member’s prompts, leading to incorrect responses to the qualitative questions posed by the app. Due to the use of a convenience sampling methodology, there was potential for positive bias and interviewer bias. Steps, including the use of structured questionnaires and predetermined simulated use procedures, were implemented to minimize this bias. Trained local interpreters were used to minimize interviewer bias. Because two villages overlapped between implementation of version I and version II of the app, there exists the possibility of study contamination between these populations; however, as the NeMo system never remained with the women beyond the interview and study members gave no indication of actions or questions being completed correctly, the risk of contamination was considered to be very low.

### Conclusions

The results of this study indicate that women are receptive to and capable of using NeMo to screen their newborns for neonatal danger signs. This study suggests that the NeMo system could be an acceptable, easy to use resource capable of expediting identification of neonatal illness to decrease delays in care-seeking behavior. While additional studies are required to assess the NeMo system’s ability to safely and effectively decrease delays in accessing neonatal care, this system, if further developed, could be an approach to decrease preventable neonatal deaths by empowering and educating mothers to detect danger signs in their newborn.

## Abbreviations

CBID: Johns Hopkins University Center for Bioengineering Innovation and Design
CGH: Johns Hopkins Center for Global Health
CHW: community health worker
GIF: graphic interchange format
IMNCI: Integrated Management of Neonatal and Childhood Illness
IQR: interquartile range
JHU-GMI: Johns Hopkins Global mHealth Initiative
LMIC: low- and middle-income country
MAKSPH IRB: Institutional Review Board of Makerere University School of Public Health
mHealth: mobile health
NeMo: Neonatal Monitoring
TBA: traditional birth assistant
UDHA: Uganda Development and Health Associates
UGX: Ugandan shilling
VHT: village health team
WHO: World Health Organization
